# Wheat lodging extraction using Improved_Unet network

**DOI:** 10.3389/fpls.2022.1009835

**Published:** 2022-09-30

**Authors:** Jun Yu, Tao Cheng, Ning Cai, Fenfang Lin, Xin-Gen Zhou, Shizhou Du, Dongyan Zhang, Gan Zhang, Dong Liang

**Affiliations:** ^1^ National Engineering Research Center for Agro-Ecological Big Data Analysis & Application, Anhui University, Hefei, China; ^2^ Key Laboratory of Geospatial Technology for Middle and Lower Yellow River Regions (Henan University), Ministry of Education, Kaifeng, China; ^3^ Plant Pathology Lab, Texas A&M AgriLife Research Center, Beaumont, TX, United States; ^4^ Institute of Crops, Anhui Academy of Agricultural Sciences, Hefei, China

**Keywords:** UAV images, Improved_Unet, deep learning, wheat, lodging extraction

## Abstract

The accurate extraction of wheat lodging areas can provide important technical support for post-disaster yield loss assessment and lodging-resistant wheat breeding. At present, wheat lodging assessment is facing the contradiction between timeliness and accuracy, and there is also a lack of effective lodging extraction methods. This study aims to propose a wheat lodging assessment method applicable to multiple Unmanned Aerial Vehicle (UAV) flight heights. The quadrotor UAV was used to collect high-definition images of wheat canopy at the grain filling and maturity stages, and the Unet network was evaluated and improved by introducing the Involution operator and Dense block module. The performance of the Improved_Unet was determined using the data collected from different flight heights, and the robustness of the improved network was verified with data from different years in two different geographical locations. The results of analyses show that (1) the Improved_Unet network was better than other networks (Segnet, Unet and DeeplabV3+ networks) evaluated in terms of segmentation accuracy, with the average improvement of each indicator being 3% and the maximum average improvement being 6%. The Improved_Unet network was more effective in extracting wheat lodging areas at the maturity stage. The four evaluation indicators, Precision, Dice, Recall, and Accuracy, were all the highest, which were 0.907, 0.929, 0.884, and 0.933, respectively; (2) the Improved_Unet network had the strongest robustness, and its Precision, Dice, Recall, and Accuracy reached 0.851, 0.892, 0.844, and 0.885, respectively, at the verification stage of using lodging data from other wheat production areas; and (3) the flight height had an influence on the lodging segmentation accuracy. The results of verification show that the 20-m flight height performed the best among the flight heights of 20, 40, 80 and 120 m evaluated, and the segmentation accuracy decreased with the increase of the flight height. The Precision, Dice, Recall, and Accuracy of the Improved_Unet changed from 0.907 to 0.845, from 0.929 to 0.864, from 0.884 to 0.841, and from 0.933 to 0.881, respectively. The results demonstrate the improved ability of the Improved-Unet to extract wheat lodging features. The proposed deep learning network can effectively extract the areas of wheat lodging, and the different height fusion models developed from this study can provide a more comprehensive reference for the automatic extraction of wheat lodging.

## 1 Introduction

Wheat is the second largest food crop in the world. Various factors, including adverse weather, disease and insect pests, high-density planting, and excessive nitrogen application, can reduce plant photosynthesis (Setter et al., 1997; Zhang et al., 1999) and lead to lodging, making it difficult for harvesting. Lodging is one of the most important factors affecting wheat yield and quality (Kobayashi and Hitaka, 1968; Rongtian et al., 1996; Cao et al., 2021). Yield reduction of up to 45% has been reported (Berry and Spink, 2012; Peng et al., 2014). Lodging poses a severe threat to global food security. China is one of the largest wheat producers. The annual wheat planting area in China is about 24,000 hectares, and the yield reduction caused by lodging is as high as 10%. At present, there is an urgent need to develop new technologies and methods that can quickly and nondestructively assess wheat lodging to minimize yield loss.

The traditional methods to monitor lodging require an agricultural technician to sample and analyze the lodging areas on site (Chu et al., 2017). When a large area of lodging occurs, the traditional detection methods are not only time-consuming but have low accuracy as well (Mardanisamani et al., 2019), which often leads to insurance compensation disputes and negatively affects the farming enthusiasm of farmers and enterprises. Remote sensing is an effective method to obtain information remotely, which can accurately assess the temporal and spatial changes of crops. Remote sensing can provide strong support for lodging assessment (Weiss et al., 2020). The commonly used remote sensing methods include satellite remote sensing, aerial remote sensing, near-earth remote sensing, etc. Satellite remote sensing can cover a large scale, which has been increasingly widely used in crop lodging extraction (Wang et al., 2016; Han et al., 2017; Kumpumäki et al., 2018). However, due to low temporal and spatial resolution and limited availability of meteorology data in southern China (frequently cloudy and rainy weather), the accuracy of satellite remote sensing in lodging recognition is low (Kustas and Norman, 2000). Also, it is challenging to meet the demand of rapid response due to the long return visit cycle of the satellite. Compared with space remote sensing, near-earth remote sensing can maneuver and respond rapidly and is suitable for monitoring lodging caused by floods. However, due to China’s airspace policy and flight costs, its scientific research and technology promotions become difficult to be carried out. Near-earth remote sensing uses a small Unmanned Aerial Vehicle (UAV) equipped with sensors to obtain various high-resolution data, such as high-definition RGB images, multispectral images, hyperspectral images, and thermal images. Its low cost, high flexibility, and strong ability to provide high spatial resolution (Alvarez-Vanhard et al., 2021) make it a hot research area in crop lodging monitoring. For example, Liu et al. (2014); Hyundong et al. (2021), and Tianxing et al. (2017) used UAVs to assess the lodging of rice, wheat, corn, and other crops. Singh et al. (2019) demonstrated through their study that large-scale quantitative assessment of inversion using a UAV is an effective method for identifying genetic variation in inversion. However, current methods to process large UAV image data have become the bottleneck for their further applications. It is in urgent need of exploring a new method to effectively process UAV lodging data.

At present, quite a few scholars have studied using UAV as new methods to assess crop lodging. Ding et al. (2019) used YCbCr transform and support vector machine to extract the wheat lodging area, with an accuracy of 92%. Liu et al. (2018) used UAV visible light combined with a thermal infrared image as classification data, combined color, texture, and temperature characteristics, and used the optimization of SVM multiclass by Particle Swarm (PSO-SVM) method to obtain a value of 0.9415 for the correlation coefficient between the lodging proportion and the actual lodging proportion. Chauhan et al. (2019) used the UAV multispectral image and adopted the multi-resolution segmentation (MRS) algorithm and nearest neighbor classification algorithm to realize the classification of lodging wheat with different severities. It is found that the red edge and near-infrared band data could effectively distinguish different severity categories, and the overall accuracy reached 90%. Cao et al. (2021) proposed a hybrid algorithm based on a watershed algorithm and adaptive threshold segmentation to extract wheat lodging, which was better than a single watershed algorithm. However, the above methods require technicians to screen and identify features, which are difficult to apply and popularize in business systems. The emergence of deep learning provides an important opportunity to solve the above problems. With the continuous research of deep learning algorithms, deep learning has made major breakthroughs in the classification, detection, and segmentation tasks (Simonyan and Zisserman, 2014; Szegedy et al., 2015; Redmon et al., 2016; Krizhevsky et al., 2017; Ren et al., 2017; Shelhamer et al., 2017). On the study of crop lodging, some investigators have made a preliminary exploration by using deep learning. Zhao et al. (2019) used Unet network to extract the lodging areas at the late mature stage of rice, and the Dice coefficient could reach 0.9442. Yang et al. (2020) used FCN-Alexnet network to train the visible image data of lodged rice, and realized the lodging extraction accuracy of 0.9443. Song et al. (2020) used the self-defined Improved_Segnet method to extract the sunflower lodging areas of the fused image, and the accuracy was 15 to 20% higher than that of the traditional support vector machine (SVM). Further, in the depth learning method of wheat lodging, Zhang et al. (2020a) used transfer learning to train the DeeplabV3+ model to extract the wheat lodging areas at different growth stages. Their results show that the effect of the transfer learning method was better than that of the traditional Unet method. Yang et al. (2021) improved Unet network for wheat lodging, with an overall accuracy of 88.99%. Zhang et al. (2020b) showed that the accuracy of UAV RGB images, combined with the GoogLeNet deep learning algorithm, could reach 0.93 based on UAV RGB data from three different collection dates, combined with different deep learning algorithms, for wheat inversion monitoring. Compared with traditional machine learning methods, the deep learning method has greater potential and space in the research of wheat lodging monitoring. However, the previous methods give consideration only to efficiency and accuracy and do not account for multiple heights (scales), which makes the algorithm difficult to be applied in practice.

With the introduction of Involution operator (Li et al., 2021a), it provides a new idea to solve the contradiction between the number of layers and the accuracy of the deep learning network. It achieves higher efficiency and higher classification accuracy without changing the overall structure of the network, which provides a new direction for UAV remote sensing monitoring of crop lodging. Furthermore, investigators often combine the front and rear characteristics to improve the performance of deep learning networks. For example, Dense block is often used for the classification and recognition of detection targets, and the way to retain the pre and post features to improve the classification accuracy has not been used for wheat lodging extraction. Therefore, it is worth trying to combine Involution operator and Dense block to develop a method to assess wheat lodging.

In view of the lack of effective methods for wheat lodging monitoring based on UAV images, the objectives of this study were to: 1) propose a new wheat lodging assessment method based on Involution operator and Dense block module at different flight altitudes, 2) verify the effectiveness of the proposed method by comparing with several classical deep learning methods, and 3) verify the robustness of the proposed method through the data collected from different flight altitudes and different wheat production areas. The research results will support the study of lodging extraction at different flight altitudes, the discrimination of actual lodging conditions of farmland, and post-disaster damage assessment.

## 2 Materials and methods

### 2.1 Experimental area and UAV data acquisition

#### 2.1.1 Experimental area

**Figure 1:**
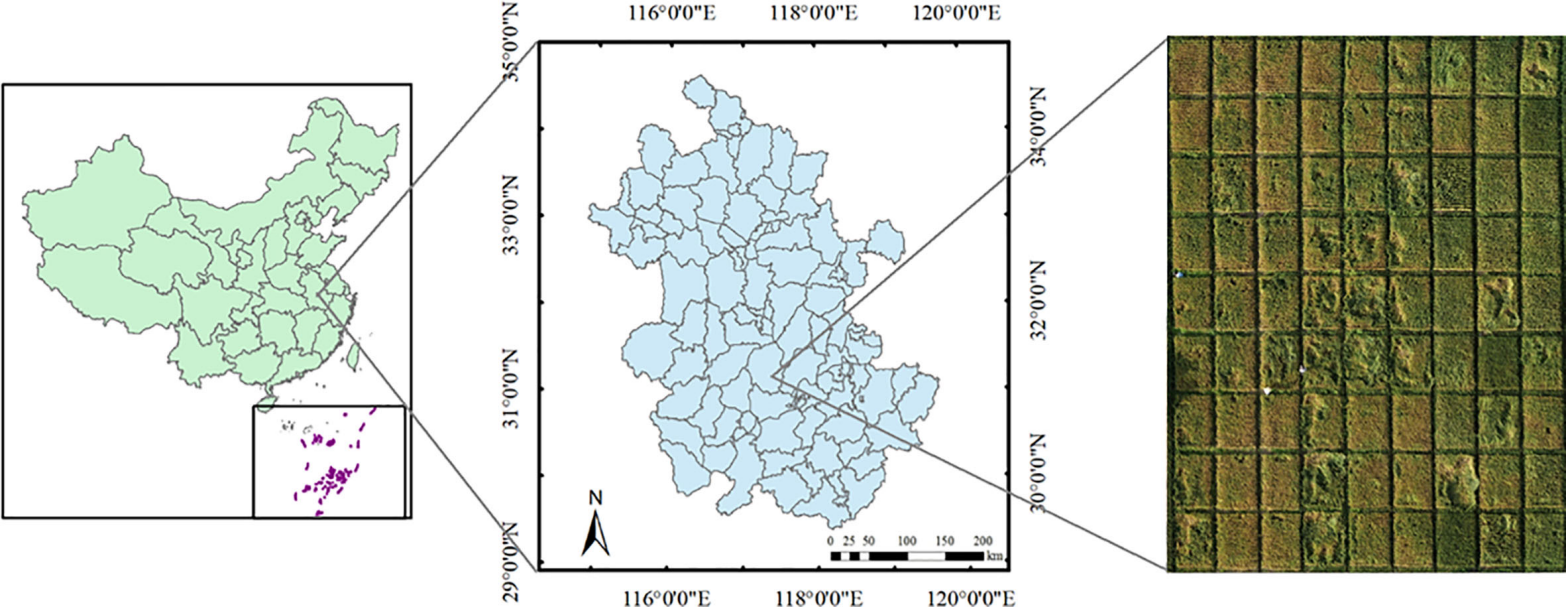
Map of the experimental area. On the left was the location of the experimental area located in Anhui Province in the East of China; in the middle was the map of Anhui Province, with the experimental field in Lujiang County in central Anhui; and on the right was the UAV lodging image of the experimental area.

The experimental area (**Figure 1**) was in Baihu farm, Lujiang County, Hefei City, Anhui Province, China (31°13’ N, 117°27’E). It was in the subtropical monsoon climate zone, with mild climate, abundant rainfall, and an average temperature of 13 to 20 °C, suitable for wheat production. Wheat was one of the most widely cultivated crops in this area.

Wheat (cv. Ningmai 13) was sown in October 2019. Field plot management such as fertilization, irrigation, and insect and weed control, etc. followed local recommendations. The growth status of wheat was different from one area to another due to the difference in soil fertility and salinity. UAV images of lodged wheat areas were collected on May 3 (filling stage) and May 10 (maturity), 2020 (**Figure 1**).

#### 2.1.2 Data acquisition

In this study, the lodging images of UAV at two flight altitudes (20 and 40 m) were collected at the grain filling and maturity stages. The observation platform was DJI Phantom 4 Pro (DJI Innovation Science and Technology Co., Shenzhen, China), and the image resolution of its own camera was 5472×3648 pixels. The UAV flights used DJI GS Pro software to plan the route, with 80% forward overlap and 80% side overlap. At the flight altitude of 20 and 40 m, the flight speeds were 2m/s and 4m/s, respectively. The UAV images were collected at 14:00 on May 3 and 10, 2020, taking about 30 minutes for each flight. The flight day was sunny and breezy. In order to improve the geographic coordinate accuracy of UAV images, Trimble R2 was used as the ground control points for geometric correction of UAV images. The UAV images were spliced with Photoscan (Agisoft, St. Petersburg, Russia) software, during which the ground control points were imported for accurate geometric correction, and finally, the Orthophoto Image of the study area was exported. **Table 1** shows the detailed information on the UAV image acquisition. In order to observe the influence of different altitudes on the extraction results of wheat lodging based on the obtained data, the UAV images were downscale sampled by cubic convolution interpolation method, and the flight altitude data of 80 and 120 m were produced.

**Table 1:** Summary of UAV image acquisition.

Date	Flight height	Speed	Photo interval	Lodging area	Spliced image size (pixels)
May 3	20 m	2 m/s	2 sec	Small	16279*11823
	40 m	4 m/s	2 sec		8049*5486
	80 m (Resampling)	/	/		/
	120 m (Resampling)	/	/		/
May 10	20 m	2 m/s	2 sec	Large	16339*11636
	40 m	4 m/s	2 sec		8055*5410
	80 m (Resampling)	/	/		/
	120 m (Resampling)	/	/		/

*Represents the multiplication sign in the mathematical operator.

**Figure 2:**
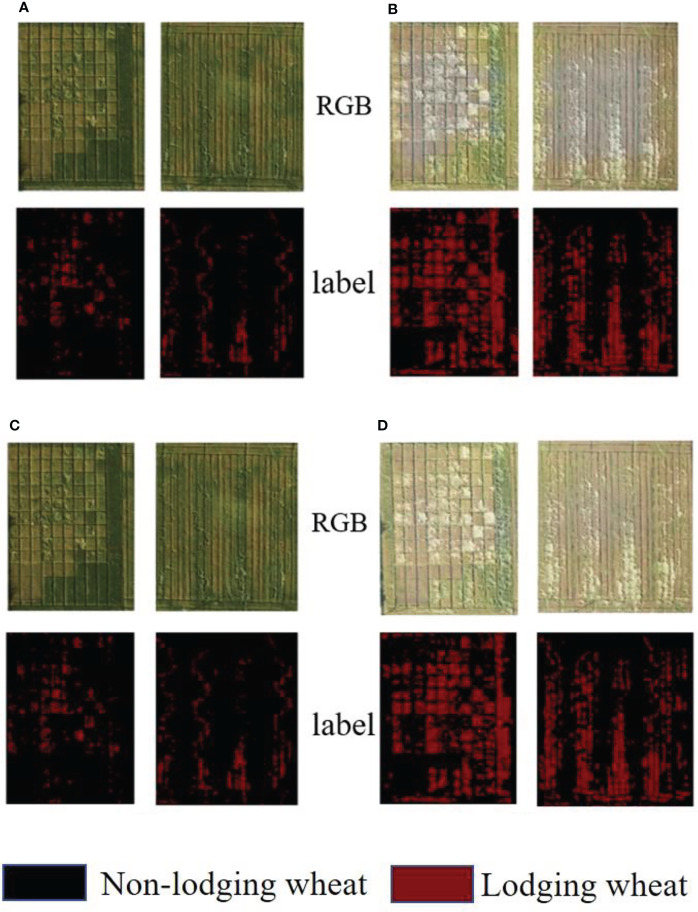
RGB original images and label images at two growth stages and two flight heights. **(A)** 20-m flight altitude (May 3), **(B)** 20-m flight altitude (May 10), **(C)** 40-m flight altitude (May 3), **(D)** 40-m flight altitude (May 10).

In order to make the data set needed for the training of the deep learning network, the Orthophoto Images were firstly cut to get the research areas, then the lodging areas in the UAV image were manually labeled with the help of agricultural experts who used the Open source software LabelMe (Massachusetts Institute of Technology CSAIL, Cambridge, MA, USA), and finally obtained the UAV RGB image and label map at the height of 20 and 40 m at the two growth stages. The results are shown in **Figure 2**. The red area was the lodging wheat label manually marked. Comparing with the labels of the two growth stages, as wheat growth advanced, the red areas were shown in the label map. The larger the area of lodging, the more serious the wheat lodging. The data images and label diagrams at the 80- and 120-m flight altitudes were not listed here. The above data was obtained from the down-sampling of 20-m flight altitude data.

### 2.2 Experimental method

The UAV image data set used for training in this study came from the four flight altitudes of 20, 40, 80, and 120 m, including multiple spatial resolution data. Some of these data were used to verify the Improved_Unet proposed in this study. The accuracy and robustness between the proposed network and other classical segmentation networks were compared.

#### 2.2.1 The proposed wheat lodging extraction Improved_Unet network

The classical Segnet network, DeeplabV3 +, and Unet network have been used in the previous studies for crop lodging extraction, and high model accuracy has been obtained. Among them, the Segnet network adopts different feature acquisition strategies than the traditional FCN network, and it uses coding and decoding technology and uses the pooled index to save pooling location information. DeeplabV3+ network structure is also composed of encoding and decoding structures. The encoding part includes Atrus Spatial Pyramid Pooling (ASPP) and improved Xception module including input, intermediate, and output stream. Some studies have used the transfer learning method to reduce the network’s requirements for the amount of wheat lodging data, but the reasonable selection of public data set is a complex problem. Unet network adopts encoding and decoding structure, and convolution adopts a valid filling method to ensure that the results do not lack context features. In the decoding stage, the feature map obtained in the encoding stage is connected with that in the decoding stage, and the pixel level classification is eventually realized through 1 * 1 convolution. Compared with the Segnet network, Unet network has a jump connection structure, which alleviates the problem of gradient disappearance in the training process to a certain extent, simplifies the model, and makes the model easier to adapt to the complex information of wheat lodging images. It also fuses the low-level and high-level information to obtain more information. Compared with DeeplabV3+, the number of layers of Unet network is much less, and the amount of training samples required and the training time are also less. In addition, there is no need to use the method of transfer learning for training, which reduces the use standard.

Considering the differences between the above network structures and considering the actual application, this study improved the lightweight network Unet to achieve the purpose of less time-consuming model training on the premise of reliable accuracy in actual production. In order to obtain a larger receptive field and retain more information to improve the segmentation accuracy, this study used the Involution operator to replace the convolution operation of the backbone position on the basis of ensuring the network structure. In the Involution design, Involution kernels are specifically located at pixels X_i,j_ corresponding to coordinates (i, j) customized but shared on the channel, and G calculates the number of groups that share the same Involution kernel per group. The Involution kernel is used to multiply and add the inputs to obtain the out feature map of Involution defined as:


(1) $$Y_{i,j,k}=\sum_{\left(u,v\right)\in \Delta_{K}}{\mathrm{i,j}}_{\mathrm{,u}}+\left[\frac{K}{2}\right],v+\left[\frac{K}{2}\right],\left[\frac{kG}{C}\right]X_{i}+u,j+v,k.$$

The shape of the Involution kernel ℌ depends on the shape of the input feature mapping. The idea is to generate Involution kernels conditioned on the original input tensor such that the output kernel is aligned with the input kernel. Here the kernel generating function is symbolized as ∅ and the function mapping for each position (i, j) is abstracted as:


(2) $$H_{i,j}=\emptyset \left(X_{{\text{Ψ}}_{I,J}}\right)=W_{1\sigma }\left(W_{0}X_{i,j}\right)$$

where Ψ_i,j_ indexes the set of pixels conditional on ℌ_i,j_; $W_{0}\in R^{\frac{C}{r}\times C}$ and $W_{1}\in R^{(K\times K\times G)\times \frac{C}{r}}$ represent 2 linear transformations that together form the bottleneck structure, with the intermediate channel dimension controlled by the reduced order ratio r for efficient processing. σ denotes the nonlinear activation function for the 2 linear transformations after batch normalization.

**Figure 3:**
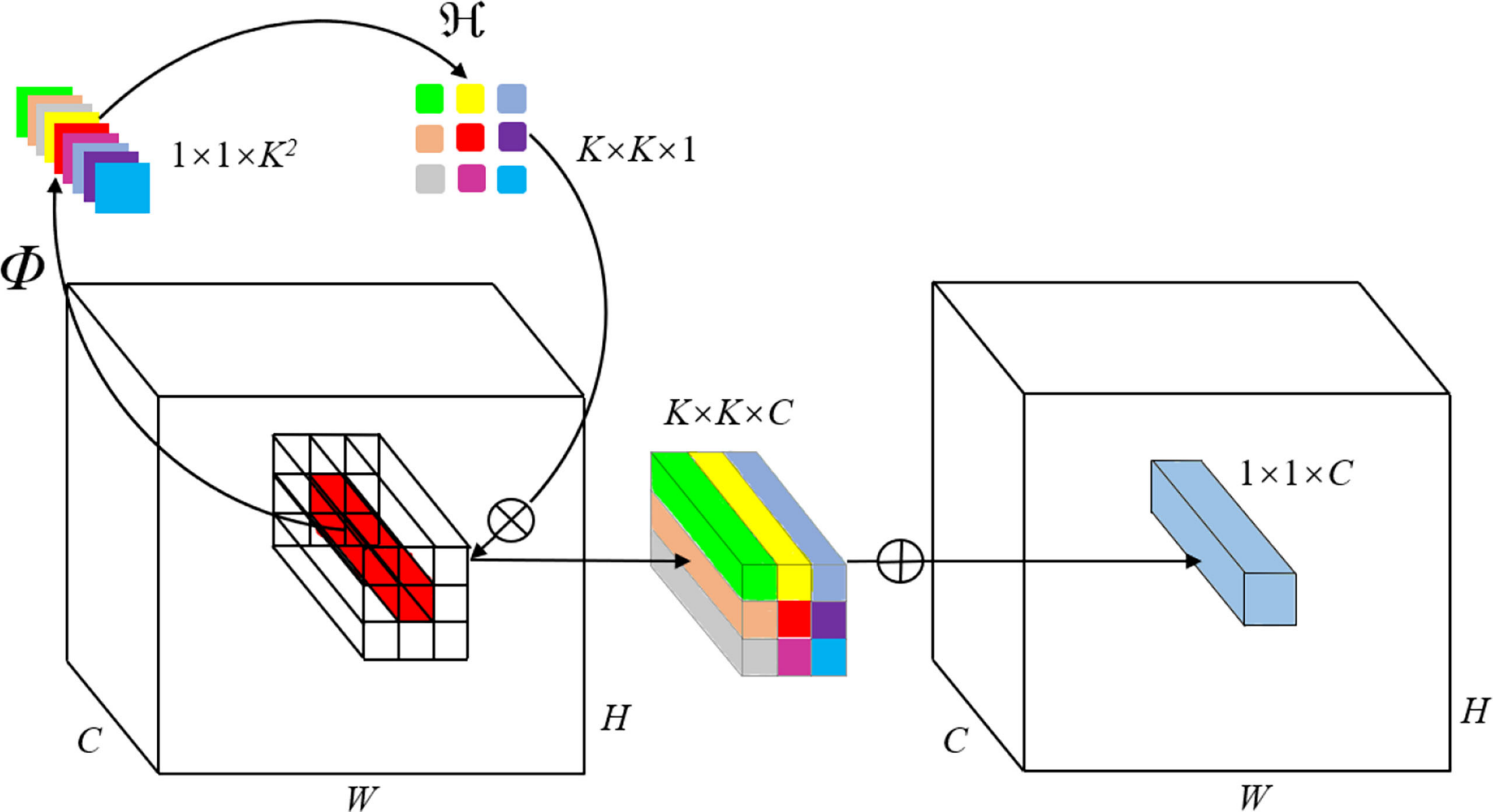
Involution diagram. Involution cleverly divides the network computation into kernel generation and Multiply-Add. The Involution kernel ℌ_i, j_ is yielded from the function Φ conditional on a single pixel at (i, j), followed by a channel-to-space rearrangement. The Multiply-Add operation of involution is decomposed into two steps, with ⊗ indicating multiplication broadcast across C channels and ⊕ indicating summation aggregated within the K × K spatial neighborhood (Yang et al., 2021).

Ordinary convolution operation realizes channel specificity: sharing convolution operators on the same channel and using different operators on different channels. The construction of the Involution operator is the opposite of convolution operation, which realizes spatial specificity, expands the receptive field based on reducing the amount of calculation, and reduces the information redundancy between channels. The diagram of Involution is shown in **Figure 3**.

ℌ is the Involution kernel, which is calculated from the function Φ. Involution uses block convolution, that is, convolution sharing on channels. For example, if the channel is 32 and 16 channels share one convolution, the block is 2. For ease of presentation, the block used in the schematic diagram is 1. Through this construction method, spatial specificity and channel sharing are realized. All 1 * 1 convolutions are retained in this study to realize classification.

At the same time, the dense block module is added in the coding stage to retain more information by combining the front and rear feature maps. DenseBlock is an important part of DenseNet network. The main idea is: for each layer, the feature maps of all previous layers serve as the input of the current layer, while its own feature maps serve as the input of the subsequent layer, forming full cross-linking. Feature maps extracted from each layer can be used by subsequent layers. Compared with other methods, DenseBlock has obvious advantages. First, the number of parameters is reduced, which can save memory and avoid model overfitting. On the other hand, it further alleviates the problem of gradient disappearance, makes more effective use of feature and enhances feature propagation.

**Figure 4:**
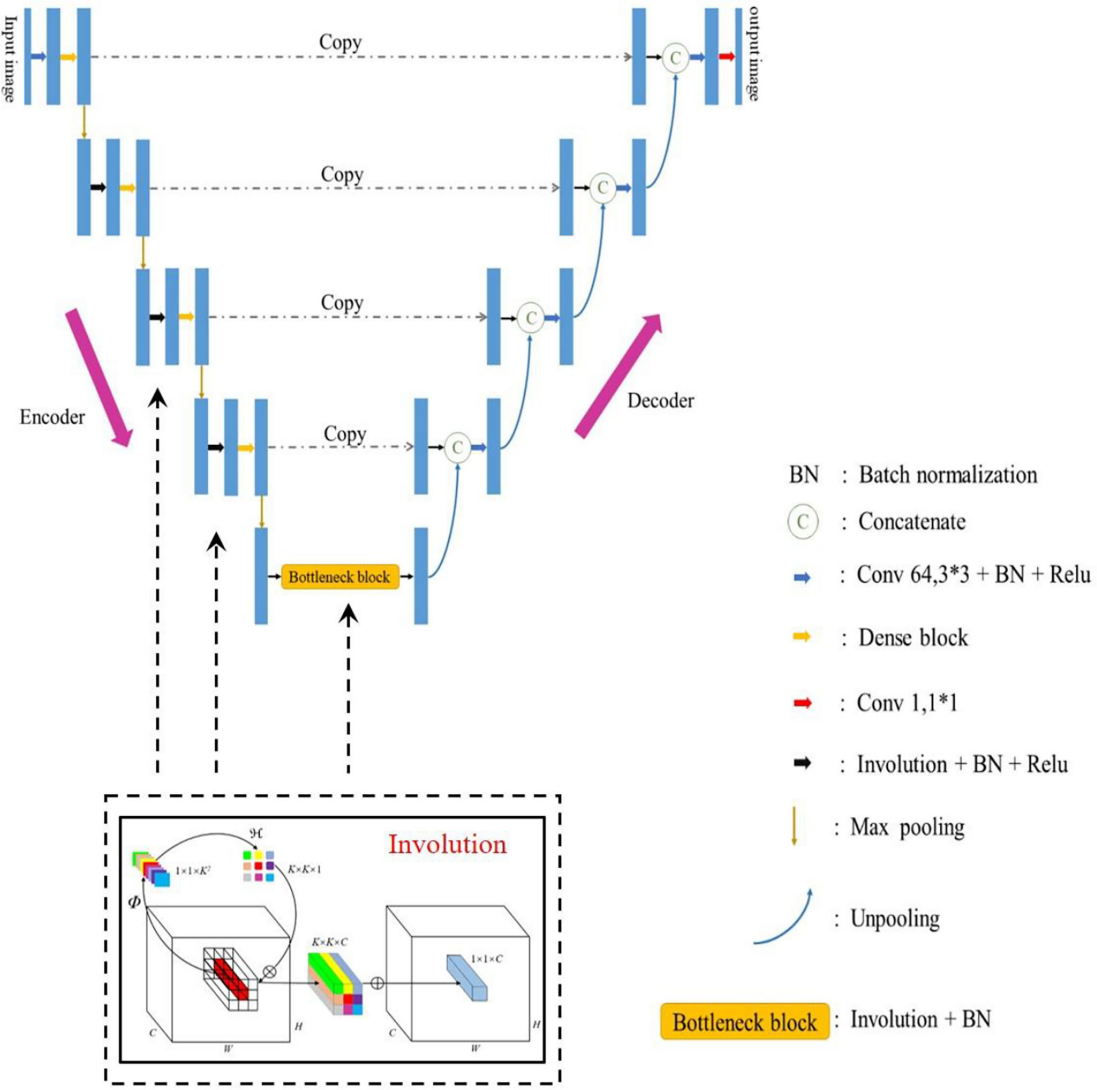
Improved_Unet network. The network was divided into Encoder and Decoder. The feature images were calculated in the Encoder part by Convolution and Involution; the Decoder part included Unpooling. The front and rear features were combined by Concatenate. The BN means Batch Normalization which speeds up the training process, improves performance and solves the problem of gradient disappearance; the C Represents Concatenate which combines front and back features; the blue arrow to the right represents Convolution 64, 3*3+BN+Relu function; the yellow arrow to the right represents Dense block who’s structure is shown in the blue box, which combines the front and rear features; the red arrow to the right represents Convolution with convolution kernel of 1*1 and number of feature maps of 1; the black arrow to the right represents Involution + BN +Relu; the Max pooling retains key features while reducing parameters; and the Unpooling enlarges image size.

Further, Max-pooling is used. During the pooling, the Max-pooling location information is retained, and then in the decoding stage, un-pooling is used to enlarge the feature map combined with the Max-pooling location information recorded during encoding. The network’s last layer uses a 1 * 1 convolution kernel and a convolution method that limits the number of feature images to achieve pixel-level classification. The Improved_Unet network structure is shown in **Figure 4**. Data set production

#### 2.2.2 Data set production

To ensure that the deep learning network obtained good training results and reduced the phenomenon of network fitting due to too little data, this study cut and widened the original UAV image data. The image was cropped by a sliding window which had size of 256*256. As a result, the size of the final training image was 256*256. The imagery data was augmented by rotating, filtering, flipping, and adding noise. The label image needed the same operation while rotating and flipping the RGB image. A total of 15,000 training images and the same number of label images were obtained. Fifteen thousand images were divided into two parts, with 12,000 images being randomly selected as the training set of the model, and the remaining 3,000 images being used as the test set of the model. The purpose of random selection was to balance the inconsistency of wheat lodging areas and light in different research plots.

#### 2.2.3 Model training

The experiment was conducted using the Windows system, Keras framework and python 3.7 programming language, with Tensorflow as the backend. Using Adam optimizer, the initial learning rate was set to 0.001 according to the original setting. The experiment was conducted in 8 g memory, Xeon E5-2630 CPU (Intel Corp., Santa Clara, USA), GeForce RTX 2080 Ti (NVIDIA Corp., Santa Clara, USA) GPU environment. Segnet network, Unet network, the batch size of the Improved_Unet network was set to 10, and the epoch was set to 100. After training, the final model was obtained. The DeeplabV3+ network using the transfer learning method used pre-trained network parameters. The parameters were obtained from the public training data set PASCAL VOC 2012. In view of the deep number of network layers, only the last layer of the network was thawed to train our own data set. Because the pretraining parameters were used, the network can be stable soon. Therefore, the epoch was set to 60 and the batch size to 10. The training environment was consistent with the previous networks. In order to balance the uneven situation of lodging and nonlodging samples, the Tversky loss function was used in all network training. In order to evaluate the obtained model, precision, dice, recall, and accuracy evaluation indexes were used to evaluate the model comprehensively. The formulas of loss function and evaluation index are shown in equations 3–7.


(3) $$TL=1-\frac{TP+\epsilon }{TP+\alpha FN+\beta FP+\epsilon }$$

(4) $$Precision=\frac{TP}{TP+FP}$$

(5) $$Dice=\frac{2TP}{2TP+FP+FN}$$

(6) $$Recall=\frac{TP}{TP+FN}$$

(7) $$Accuracy=\frac{TP+TN}{TP+TN+FP+FN}$$

Where TP (true positive) indicates the correct classification of samples (the lodging area is divided into the lodging area); FP (false positive) indicates false alarm, that is, the negative sample is divided into positive samples (the nonlodging area is divided into the lodging area); and FN (false negative) indicates false alarm, that is, the positive sample is divided into negative samples (the lodging area is divided into the nonlodging area). TL (Tversky Loss function) serves as the network loss function, and α and β represent the two hyperparameters (taking values between 0 and 1,*α* = 1-*β*). By adjusting α and β, the trade-off between false positives (False positives) and false negatives (False negatives) can be controlled. ϵrepresents the constant.

#### 2.2.4 Model validation

The Involution operator enhanced the ability to capture information. In order to further verify the robustness and segmentation accuracy of the Improved_Unet, the evaluation was also performed on the UAV images of wheat lodging collected from other farms in Shucheng County, Anhui Province, China in 2019. The image acquisition platform was DJI Elf 4pro. The shooting day was on May 10, 2019. The flight altitude was 20 m. The data processing process was the same as in Section 2.1.2. The segmentation accuracy of Improved_Unet, Unet, Segnet and DeeplabV3+ networks on the new data was used to evaluate the robustness of the constructed model.

## 3 Results

### 3.1 The effect of the proposed Improved_Unet model

**Figure 5:**
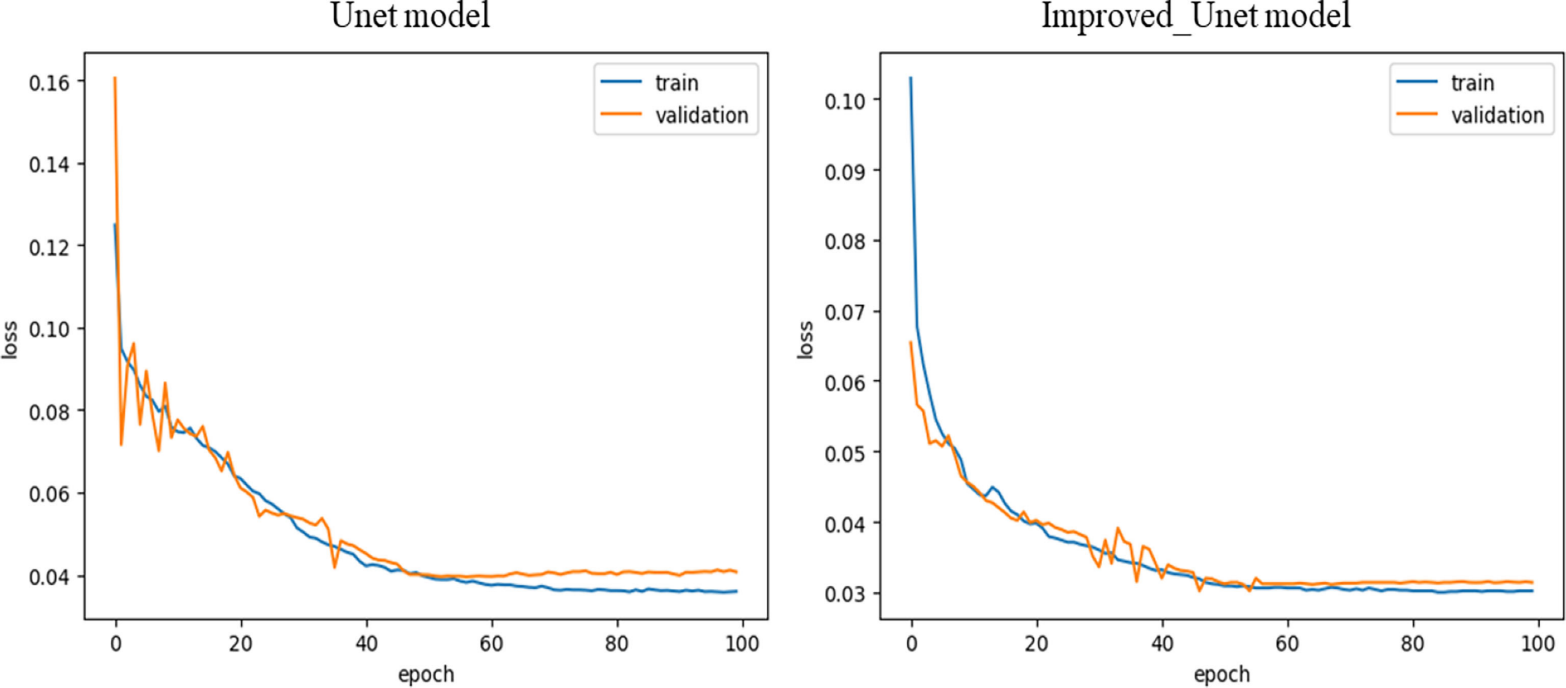
Comparison of the loss convergence curves between the Unet and Improved_Unet models.

In order to evaluate the effects of the improved network proposed in this study and its original network, the prediction accuracy of the two networks was compared after arbitrarily cropping the lodging image. **Figure 5** shows the loss convergence curves of the Unet network and the proposed Improved_Unet network in this study.

**Figure 6:**
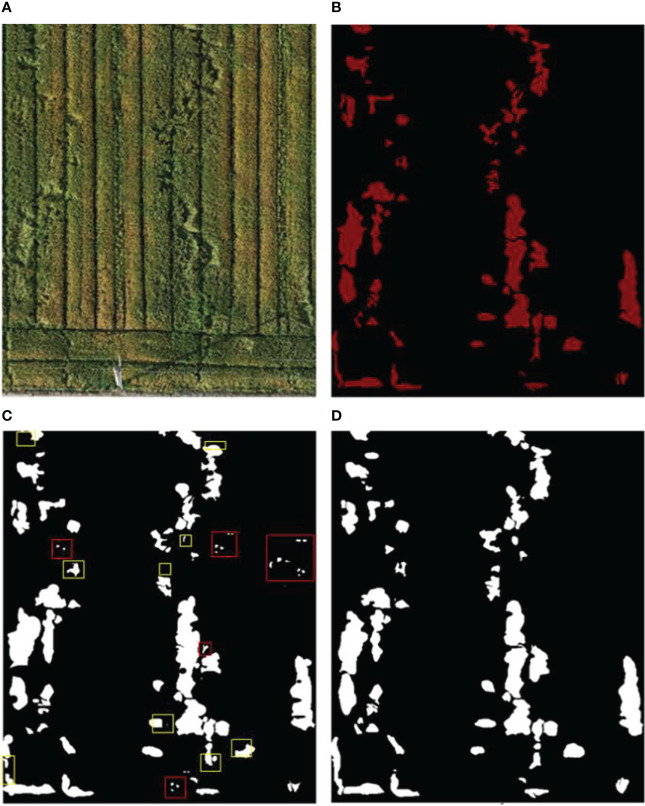
The arbitrarily cropped image of the experimental area and the prediction results of the two network models. **(A)** Original image of any site in the experimental area, **(B)** Manually labeled image, **(C)** Unet prediction results, **(D)** Improved_Unet prediction results.

As shown in **Figure 5**, compared with the Unet model, the Improved_Unet model had a faster loss convergence speed and a smoother curve. The loss of the Unet model fluctuated violently at the beginning, and the loss of the Improved_Unet model changed more gently. Especially in the interval where the loss changed less, the training of the Improved_Unet model was closer to the validation loss, and the curve fluctuation was smaller, which indicates that the Improved_Unet model is more stable. To further verify the accuracy of the proposed Improved_Unet model, the image of the study area was arbitrarily cropped, and the effects of the network before and after the improvement were compared in this study. The results are shown in **Figure 6**.

In **Figure 6**, based on the overall prediction and segmentation effect, the segmentation results of the Improved_Unet network were better in the segmentation of large and small lodging areas, and the results were closer to those manually labeled by experts. The segmentation results of the Unet network had some noise points (misclassification, as shown in the red box in **Figure 6C**), and the prediction of some small lodging sites was unsuccessful, or the prediction had a more significant deviation (as shown in the yellow box in **Figure 6C**). In order to show the results of verification analysis in a more detailed manner for the two networks, the 20-m flight height data was taken for further illustration. The results are shown in **Table 2**.

**Table 2:** Comparison of the results of four networks at the 20-m height.

Evaluation Indicators	Unet		Improved_Unet		Segnet		DeeplabV3+	
	Filling	Maturity	Filling	Maturity	Filling	Maturity	Filling	Maturity
Precision	0.870	0.875	0.892	0.907	0.821	0.837	0.881	0.865
Dice	0.881	0.894	0.918	0.929	0.868	0.877	0.901	0.913
Recall	0.872	0.876	0.879	0.884	0.854	0.863	0.870	0.881
Accuracy	0.910	0.914	0.923	0.933	0.883	0.890	0.914	0.917

After all the data training in **Table 1**, the data at a height of 20 m were separately extracted for prediction.

It can be seen from **Table 2** that the results of the four evaluation indicators of the Improved_Unet network were better than those of the Unet network, and the Accuracy and Dice coefficients of the Improved_Unet network were both greater than 0.923, achieving better segmentation results. In particular, the Accuracy at Maturity was 0.933, which was the highest among all results.

### 3.2 Accuracy comparison between the Improved_Unet network and other classical networks

To further compare the performance of the Improved_unet network with the Segnet network, Unet network and Deeplabv3+ network on lodging segmentation, the results are shown in **Table 2**. Taking the 20-m flight height data as an example, the evaluation results of Precision, Dice, Recall, and Accuracy of the four network models of Unet, Improved_unet, Segnet, and Deeplabv3+ at the two growth stages (filling and maturity) of wheat are listed. It can be seen from **Table 2** that the Improved_Unet network proposed in this study had the highest Indicators at the stages. Followed by the DeeplabV3+ network, its maturity Accuracy and Dice coefficients were 0.917 and 0.913, respectively. Overall, the method proposed in this study was superior to the other three classical segmentation networks in the extraction accuracy of lodging areas.

### 3.3 Robustness verification of the Improved_Unet in other study areas

**Figure 7:**
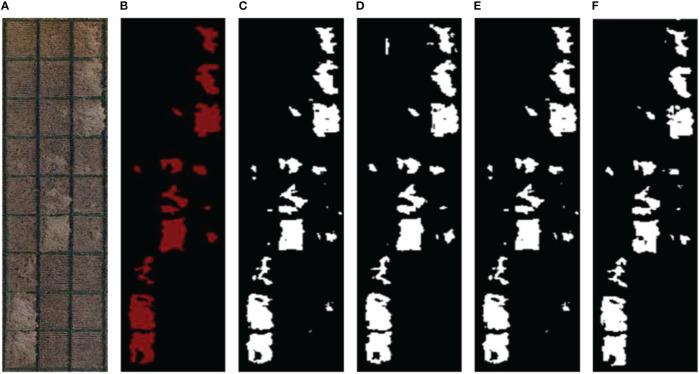
Comparison of processing results of different network models in other experimental areas. **(A)** Original image of lodging wheat taken by UAV, **(B)** Wheat lodging labeled image marked by experts, **(C)** Lodging prediction image of Unet, **(D)** Lodging prediction image of Improved_Unet, **(E)** Lodging prediction image of Segnet, and **(F)** Lodging prediction image of Deeplabv3+.

Although the Improved_Unet network model proposed in this study had higher accuracy compared to other classic network models, its robustness (whether it was still applicable in different years and different geographical regions) needed to be verified. **Figure 7** shows the arbitrarily cropping the original and manual labeled images and four kinds of network processing in the study area obtained in 2019. Specifically, **Figure 7A** was the original image of lodging wheat taken by UAV, and **Figure 7B** was the result of manual labeling by experts. **Figures 7C–F** were the prediction results of the Improved_Unet, Segnet, Unet, and DeeplabV3+ networks, respectively. **Table 3** displays the robustness verification results of the different networks.

**Table 3:** Robustness verification of different networks.

Method	Precision	Dice	Recall	Accuracy
Segnet	0.768	0.823	0.802	0.840
Unet	0.812	0.855	0.827	0.861
Improved_Unet	0.851	0.892	0.844	0.885
DeeplabV3+	0.827	0.874	0.816	0.869

**Table 3** was the prediction result of **Figure 7** after all data training in **Table 1**.

From the visual results in **Figure 7**, the lodging wheat area segmented by the Improved_Unet network was highly consistent with the results labeled by the experts. Whether it was a large lodging area or a small lodging area, the segmentation results had a high similarity with the labels. Further, the segmentation results were quantified and the results of the four evaluation indicators Precision, Dice, Recall, and Accuracy were compared. On average, each metric was improved by more than 3 percentage points, and among the individual metrics, the Precison of Improved_Unet showed the significant improvement of 10.8% over the Precison of Segnet network. It can be seen from **Table 3** that the segmentation effect of the Improved_Unet network proposed in this study was significantly better than the other three networks, and the results of the four indicators were 0.851, 0.892, 0.844, and 0.885, respectively. Among them, the segmentation result of the Segnet network was the worst. The Precision was 0.768, the Accuracy was 0.840, and the noise and misclassification were the most serious. The Precision and Accuracy of the Improved_Unet were 0.083 and 0.045 higher than those of Segnet, respectively. Therefore, the method proposed in this study had the best effect on the prediction results of these networks evaluated. Although there was little noise and misclassification, the overall segmentation results had smooth edges, and the robustness of the model verified by data in different years and geographical locations was acceptable.

## 4 Discussion

### 4.1 The difference between the proposed method and other classical networks

This study investigated the application of a deep learning algorithm in wheat lodging segmentation. Compared with the classical Segnet, Unet and Deeplabv3+ networks, the improved_Unet developed in this study has better performance on the four evaluation indicators, Precision, Dice, Recall, and Accuracy. The average improvement of each indicator was 3% and the maximum average improvement was 6%. Furthermore, the segmentation effect was also the best based on the validation tests of different years and geographical locations. It is proved that the proposed method is more accurate and more applicable. Therefore, it has more advantages in the extraction of wheat lodging. Compared with the four kinds of deep learning networks, Segnet has improved the full convolution and the information extraction method with the new encoding and decoding form (Badrinarayanan et al., 2017). However, it loses much important information in the process of convolution through the Unpooling to retain some features. It is not enough to improve segmentation accuracy. The results of **Table 3** show that the Precision of this method was less than 0.84 at the grain filling and maturity stages, and the four evaluation indexes were less than 0.84 in other areas, which was the worst among the four deep learning networks. The Unet network can improve the encoding and decoding form, the relative character of the coding part is retained by connecting the encoding and decoding features (Chen et al., 2019). Compared with the Segnet network, the accuracy was improved, the Precision, Dice, Recall and Accuracy were all higher than 0.85 at the filling and mature stages. However, the segmentation accuracy was still on average. Chauhan et al. (2019) used the UAV multispectral image and adopted the multi-resolution segmentation (MRS) algorithm and nearest neighbor classification algorithm to realize the classification of lodging wheat with different severities with an overall accuracy of 90%. Li et al. (2021b) combined a random forest with a Segnet and Unet network to cut down sunflowers, yielding an accuracy of 88%. Yang et al. (2020) obtained an accuracy of 0.8 for Segnet segmentation of rice lodging. Compared with the previous research, the proposed method has obtained a higher precision. Cao et al. (2021) proposed a hybrid algorithm based on a watershed algorithm and adap-tive threshold segmentation to extract wheat lodging, which is better than a single watershed algorithm with an accuracy of 94%. However, the method is complex in data acquisition and requires the integration of multiple algorithms for the process, which makes it difficult to be promoted for practical application in operational systems. In this study, we only need to provide RGB data and combine with the Improved_Unet network to obtain high-precision inverted monitoring. For the DeeplabV3+ network, the coding part added Atrous spatial pyramid pooling (ASPP) and the improved Xception module and retained more details, which enabled the segmentation accuracy to improve significantly (Chen et al., 2018). Above all, the accuracy achieved more than 0.9 at the filling and mature stages. Kim et al. (2021) constructed DeepLabV3 semantic segmentation model based on ResNetV2 101 backbone network to segment rice lodging with a precision of 87%, which is similar to the result of the current study. However, the network has too many layers, the training time is too long, and it can only be applied by means of migration learning in small sample data sets, which dramatically limits the scope and effect of the network. In this study, the proposed Improved_Unet combines the advantages of Segnet and Unet, uses an involution operator to replace the convolution operation of the backbone part, realizes the spatial specificity, takes different operations for different pixels, and ensures the maximum information extraction, and thus the results of the four evaluation indexes are better than those of the other three classical deep learning networks. However, when taking the 2019 validation data as an example to compare the Segnet, Unet, Deeplabv3+ and Improved_Unet networks for the average training time, it was found that the average training time of these four networks was 1.6, 1.92, 2.78 and 2.38 h, respectively. The results indicate that the training time of the method proposed in this study was relatively longer. This might be due to the fact that the combination of features before and after the coding part retains more relevant features and the number of network layers is deeper. In the follow-up research, we will focus on reducing the number of network layers to ensure more practical promotion value.

### 4.2 The effectiveness analysis of the proposed method at different flight altitudes

At present, most of the methods proposed by previous investigators are based on the single flight altitude data. Yang et al. (2021) obtained 89% accuracy of wheat lodging extraction using 30-m flight altitude data through the improved Unet model. Zhang et al. (2020a) obtained 20-m flight height image of wheat lodging for segmentation, the segmentation accuracy was more than 85%. Some scholars have also studied the UAV data for different flight heights. Zhang et al. (2022) combined three flight heights of 15, 45 and 91m in their study for wheat lodging monitoring with an accuracy of 67%. Compared with this study, we find that the Improved model, constructed by multi-height images, has similar effect on single-height lodging wheat as that constructed by other deep learning methods, but it is also suitable for multi-height estimation, which shows the advantage of application and popularization. Considering the factors such as monitoring area, operating time, and weather conditions during the operation that will impact UAV monitoring crop lodging, the flight altitude is the key to solving the above problems. Therefore, the study of flight altitude is of great significance in crop production. In this study, we collected the data of UAV images at the maturity stage as an example to discuss the adaptability and influence factors of the proposed method at four flight altitudes. The results are shown in **Table 4**.

**Table 4:** Comparison of the results of our proposed method at four different flight altitudes.

Flight height	Precision	Dice	Recall	Accuracy
20 m	0.907	0.929	0.884	0.933
40 m	0.900	0.918	0.871	0.922
80 m	0.860	0.887	0.852	0.896
120 m	0.845	0.864	0.841	0.881

**Table 4** shows that the segmentation accuracy of wheat lodging decreased with the increase of flying height from 20 to 120 m. Precision, Dice, Recall, and Accuracy were 0.907, 0.929, 0.884 and 0.933 at the 20-m flight altitude, respectively, which were the highest at all four flight altitudes. The 120-m flight altitude was the worst, and its four indexes decreased from 4 to 7%. Therefore, it can be concluded that different flying heights affect the precision of wheat lodging segmentation. However, further analysis of the results shows that there was no significant difference in the results of lodging segmentation when comparisons were made between 20 and 40 m and between 80 and 120 m, and the difference of the four precision indexes was small, with the ranges from 0.007 to 0.013 and from 0.011 to 0.023, respectively (**Table 4**). When the flight altitude changed from 40 to 80 m, the difference between the four indexes of the lodging segmentation precision was between 0.019 and 0.031, which was obviously increased. The possible reasons are as follows: (1) with the increase of flying altitude, the larger the study area in the original image, the more ground objects (bare soil, weeds, etc.) are contained in a single pixel, and the less detailed information of the plant to some extent. In such way, it interferes with the accuracy of the study; and 2) the data of 80 and 120 m were sampled from the 20-m altitude, and there might be some loss of information (Xiao et al., 2020). The Peak Signal to Noise Ratio (PSNR) values of the original and down-sampling images were 32DB and 27DB, respectively. The higher the altitude, the more information may be lost. To sum up, the determination of effective flight altitude is beneficial to maximizing UAV’s operational efficiency and achieving reasonable lodging segmentation precision.

### 4.3 Future work

In this study, both the grain-filling and maturity stages were studied. Wheat lodging usually is more severe at these stages than any earlier growth stages. Therefore, the proposed Improved_Unet network has a good segmentation effect and can provide technical support for lodging estimation. However, developing a method to monitor lodging at the early and middle stages of wheat growth and development can help take preventative farming measures to reduce the yield loss caused by lodging. This could be one of the research areas in the future studies.

## 5 Conclusions

With a focus on wheat lodging extraction in the current study, we collected the UAV RGB images of wheat at the filling and maturity stages using DJI Phantom 4Pro flying at different flight altitudes. Through data processing and analysis, and model accuracy evaluation, we have developed the lodging extraction network Improved_Unet with good applicability and robustness. This new method for wheat lodging extraction is an improvement over the Unet network. The Improved_Unet network has the best results in the four evaluation indexes compared with Segnet network, Unet network and DeeplabV3+ network. Taking the 20-m flight altitude as an example, the values of Accuracy and Dice in the evaluation indexes were both greater than 0.915, and the segmentation result was the best. For the combined data from both two growth stages, the Accuracy of the maturity stage was 0.933, which was better than that of the filling stage. The results were verified by using data from different flight altitudes and found that the 20-m flight altitude was the best. With the increase of flight altitude, the segmentation accuracy of wheat lodging decreased successively. Although the research method proposed in this paper has high accuracy in extracting wheat lodging, its practical application needs to be further studied in the future.

## Data availability statement

The raw data supporting the conclusions of this article will be made available by the authors, without undue reservation.

## Author contributions

Conceptualization, JY and NC; methodology, JY, TC and NC; software, NC; validation, JY, TC, NC and DZ; formal analysis, XGZ; data curation, FL; writing—original draft preparation, JY, TC and NC; writing—review and editing, JY, TC, NC, X-GZ, FL, SD, DZ and GZ; visualization, JY, TC and NC; supervision,DL, and DZ; project administration, DZ; funding acquisition, DZ. All authors agree to be accountable for the content of the work. All authors contributed to the article and approved the submitted version.

## Funding

The work was funded by the Key Research and Technology Development Projects of Anhui Province (Grant No. 202004a06020045), the Anhui Provincial Agricultural Science and Technology Achievements Project: (Grant No. 2021ZH002) and the Open Fund of Key Laboratory of Geospatial Technology for the Middle and Lower Yellow River Regions (Henan University), Ministry of Education (Grant No.GTYR202104 ).

## Acknowledgments

The authors express their sincere thanks to the people who aided this work and provided the data used in this article, and acknowledge the valuable suggestions provided by peer reviewers.

## Conflict of interest

The authors declare that the research was conducted in the absence of any commercial or financial relationships that could be construed as a potential conflict of interest.

## Publisher’s note

All claims expressed in this article are solely those of the authors and do not necessarily represent those of their affiliated organizations, or those of the publisher, the editors and the reviewers. Any product that may be evaluated in this article, or claim that may be made by its manufacturer, is not guaranteed or endorsed by the publisher.
